# Extraction-free LAMP-LFD assay for early diagnosis of congenital Chagas disease directly from neonatal dried blood spots

**DOI:** 10.3389/fcimb.2026.1934037

**Published:** 2026-10-01

**Authors:** Luciana Larocca, Fabiana G. Stolowicz, Matilde Abudara, Esteban L. Figueroa, Gustavo Maccallini, Santiago Werbajh, Adrian A. Vojnov, Claudio Aranda, Carolina Carrillo

**Affiliations:** 1 Instituto de Ciencia y Tecnología “Dr. César Milstein”, Consejo Nacional de Investigaciones Científicas y Técnicas (CONICET) - Fundación Cassará, Buenos Aires, Argentina; 2 Laboratorio Central, Hospital General de Agudos “Dr. Carlos G. Durand”, Buenos Aires, Argentina; 3 Administración Nacional de Laboratorios e Institutos de Salud (ANLIS) “Dr. Carlos Malbrán”, Ministerio de Salud de la Nación, Buenos Aires, Argentina; 4 Facultad de Medicina, Universidad Católica Argentina, Buenos Aires, Argentina

**Keywords:** Chagas disease, dried blood spots, lateral flow assay, loop-mediated isothermal amplification, newborn screening, point-of-care diagnostics, *Trypanosoma cruzi*

## Abstract

**Background:**

Vertical transmission is a major driver of new Chagas disease cases. Early diagnosis in newborns is essential for effective treatment; however, conventional screening limitations and high patient attrition lead to severe under-registration. Simplified molecular tools can substantially improve early detection and treatment access.

**Methodology/principal findings:**

We developed a simplified loop-mediated isothermal amplification (LAMP)-based assay targeting the repetitive satellite DNA of *Trypanosoma cruzi* for qualitative detection in newborn dried blood spots (DBS). Tailored for resource-limited settings, the protocol requires only a dry bath incubator, utilizing lateral flow dipsticks (LFD) for visual readout. The DBS sample is loaded directly into the reaction without nucleic acid extraction. Using artificially spiked DBS, the limit of detection was established at 1 fg of DNA and 1 parasite per reaction. Inclusivity was confirmed across all six *T. cruzi* discrete typing units (DTUs TcI–TcVI), with no cross-reactivity against heterologous or human DNA. A clinical proof-of-concept evaluation on a blinded panel of 24 newborn DBS samples (4 positive, 20 negative) achieved 100% concordance with definitive clinical status. Notably, the assay successfully identified all 4 congenital cases, including 3 that had tested negative by conventional parasitological microscopy at birth.

**Conclusions/significance:**

This simplified, extraction-free molecular test requires minimal equipment and is fully compatible with routine newborn screening infrastructure. It provides a robust, translatable tool for early congenital Chagas diagnosis in decentralized healthcare settings, with high potential to improve detection rates and secure timely access to etiological treatment.

## 1 Introduction

Chagas disease, caused by the protozoan *Trypanosoma cruzi*, is a complex socio-environmental health problem (Sanmartino et al., 2019) that remains a critical public health challenge globally, with an estimated 7–8 million people currently infected worldwide (WHO, 2023). Vertical (congenital) transmission accounts for a substantial and growing proportion of new cases annually, causing at least 15,000 new infections each year in Latin America (PAHO, 2017) and positioning it as a primary route of *T. cruzi* dissemination across both endemic and non-endemic territories (Carlier et al., 2019). In Argentina, approximately 1,000 babies are born with Chagas each year; yet, diagnostic coverage within the neonatal population remains critically limited (Ministerio de Salud de la República Argentina, 2022).

Early diagnosis of congenital infection is essential since trypanocidal treatment administered during the neonatal period is highly effective, well tolerated, and associated with cure rates substantially exceeding those achieved later in life (Sosa-Estani et al., 2012). However, infected newborns are frequently asymptomatic at birth (Freilij and Altcheh, 1995); this absence of clinical warning signs severely hinders early diagnosis. Compounding this challenge, current diagnostic methods, organised in multi-step screening protocols, present significant barriers to effective detection: parasitological microscopy techniques require experienced personnel and may fail to detect low or fluctuating parasitemia; serological assays are unreliable in early infancy due to the persistence of maternally transferred antibodies; and molecular methods such as qPCR offer high sensitivity but their implementation in routine clinical settings remains limited due to infrastructure requirements, cost, and technical complexity, particularly in resource-constrained environments (Bern, 2015; Carlier et al., 2019). Indeed, national epidemiological data reveals that loss to follow-up for exposed infants exceeds 78% nationwide, escalating to over 95% in high-endemic regions (Ministerio de Salud de la República Argentina, Dirección de Epidemiología, 2025a), with similar evidence at regional level (Messenger et al., 2017). Consequently, there is an urgent need for decentralised, point-of-care diagnostic tools capable of shortening the time-to-result and ensuring early therapeutic intervention directly at the point of care.

Loop-mediated isothermal amplification (LAMP) has emerged as a promising molecular diagnostic approach (Notomi et al., 2000), combining high analytical sensitivity with operational simplicity. As the LAMP reaction runs at constant temperature, it can be performed using a simple heating device; results can be obtained rapidly and readout systems easily interpreted in low-complexity settings. Previous studies have demonstrated the feasibility of LAMP-based detection of *T. cruzi* DNA in different clinical matrices, including liquid blood and dried blood spots, though most approaches still require a prior nucleic acid extraction step (Rivero et al., 2017; Besuschio et al., 2020; Longhi et al., 2023).

Argentina provides a particularly compelling context for the implementation of simplified neonatal molecular diagnostics. The National Newborn Screening Law (No. 26.279) mandates universal newborn screening, generating a well-established framework for dried blood spot (DBS) sample collection on neonatal screening cards across the country. Additionally, the National Chagas Law (No. 26.281) establishes the right of all children born to *T. cruzi*-seropositive mothers, or born to mothers with undetermined status, to receive Chagas disease screening, under a multi-step algorithm (Ministerio de Salud de la República Argentina, 2022, 2025b). Together, these legal frameworks create both a public health obligation and a logistical opportunity: DBS samples are already being collected systematically from at-risk newborns, but the molecular tools needed to fully exploit this opportunity for congenital Chagas diagnosis remain limited.

Here we report the development, analytical validation, and clinical proof-of-concept of a LAMP-LFD test for the qualitative detection of *T. cruzi* satellite DNA directly from neonatal DBS samples without prior nucleic acid extraction. This approach was designed to be extremely simple—requiring only a single heating device—while achieving a robust analytical and promising preliminary clinical performance compatible with routine newborn screening programs.

## 2 Materials and methods

### 2.1 Assay design, components and steps

The developed molecular kit integrates all reagents required for isothermal amplification and visual readout within pre-formulated, calibrated dropper bottles (30 µL drops). This ready-to-use, pipetting-free configuration was designed to minimize manual handling errors and mitigate amplicon carryover contamination in low-complexity settings.

Dropper bottle 1 (Db1) consists of a stabilized master mix containing *Bst* 2.0 DNA Polymerase (New England Biolabs, USA), dNTPs (InBio Highway, Argentina), and a proprietary optimized concentration of non-ionic detergents and betaine (Merck, Germany) in a saline reaction buffer. This formulation, combining a highly processive, inhibitor-tolerant polymerase with optimized non-ionic detergents and betaine, is designed to counteract common inhibitors present in dried blood spot eluates -including hemoglobin, IgG, and anticoagulant salts- enabling direct amplification without a dedicated nucleic acid extraction or purification step, consistent with strategies previously applied to other extraction-free LAMP protocols on complex blood matrices (Rivero et al., 2017; Besuschio et al., 2020). Dropper bottle 2 (Db2) contains the specific *T. cruzi* LAMP primer set targeting the highly repetitive satellite DNA sequence; all oligonucleotides were synthesized according to the sequences detailed in **Supplementary Table S1**. Dropper bottle 3 (Db3) contains a saline developer solution for the simplified visual chromatographic readout on lateral flow dipsticks (LFD) (Milenia Biotec GmbH, Germany). The exact buffer formulations and reaction molarities are subject to commercial confidentiality guidelines, as the technology (CHAGAS NEOKIT TecnoAMI) has been transferred to Laboratorio Pablo Cassará SRL (Argentina) for industrial scaling and regulatory manufacturing compliance.

LAMP primer sets were designed using LAMP Designer software (PREMIER Biosoft International, Palo Alto, CA, USA), which generated all six primers per target (F3, B3, FIP, BIP, LF, LB) for each candidate sequence. Primer sets targeted different sequences of *Trypanosoma cruzi* genome, including two non-overlapping regions of the kinetoplast DNA (kDNA) minicircle conserved fragment, the 24S-alpha, arginine kinase, a conserved hypothetical protein and the satellite DNA (Sat-DNA) sequence. All primers were synthesized and PAGE-purified by Macrogen Inc. (Seoul, South Korea).

Following the design of multiple candidate primer sets, preliminary analytical assays were conducted to evaluate their analytical sensitivity and specificity. Based on these screening results, the Sat-DNA primer set was selected for definitive assay optimization. This sequence (GenBank accession numbers AY519985- AY520098), a highly repetitive region of approximately 195 bp present in 10^4^ - 10^5^ copies per haploid genome (Gonzalez et al., 1984; Elias et al., 2003), is conserved across all six *T. cruzi* discrete typing units (DTUs TcI–TcVI) (Zingales et al., 2009; Brenière et al., 2016; Besuschio et al., 2017, Besuschio et al., 2020). The selected primer set comprises six specific oligonucleotides: the forward and backward outer primers (F3 and B3), the forward and backward inner primers (FIP and BIP), and the forward and backward loop primers (FL and BL) (**Supplementary Table S1**). The FIP primer was labeled with biotin at its 5’ end, and the BIP primer was labeled with FAM (fluorescein amidite) at its 5’ end to enable a simplified, qualitative, naked-eye readout via LFD. During lateral flow migration, the FAM moiety of the dual-labeled amplicon is first captured by an anti-FAM antibody conjugated to colloidal gold, forming a mobile immunocomplex; this complex is subsequently immobilized at the test line by streptavidin, which binds the incorporated biotin label, while unbound gold conjugate is captured at the control line by a secondary anti-species antibody, generating the corresponding visual signals.

For analytical assays, the sample consisted of 5 µL of purified DNA, 5 µL of trypomastigote suspension, or a 5.9 mm diameter disc (approximately 15 µL) punched from whole blood spiked with DNA or trypomastigotes and dried onto Whatman or Schleicher & Schuell 903 neonatal screening filter paper. For clinical evaluations, the sample consisted of a 5.9 mm disc punched from a dried blood spot (DBS) on a neonatal screening card. For all sample types, the 5 µL liquid volume or the punched paper disc was introduced directly into a reaction tube containing one drop each of Db1 (enzyme mix) and Db2 (primer solution).

Following the optimization of reaction temperatures and reagent concentrations to maximize analytical performance, isothermal amplification was established at a constant temperature of 64 °C for 60 minutes using a dry bath incubator as the sole required equipment. During amplification, the simultaneous incorporation of both modified primers yielded dual-labeled amplicons, enabling rapid visual interpretation via an immunochromatographic LFD assay.

After incubation, amplification products were initially visualized by two independent methods to verify LFD performance: (i) agarose gel electrophoresis [1.5% w/v agarose in 1x TAE buffer, run at 50 V for 60 minutes, stained with SYBR Safe (Thermo Scientific, Waltham, MA, USA)]; and (ii) LFD readout. Upon confirming full concordance between both detection methods, subsequent assays were performed exclusively via LFD. For this latter readout, the reaction tube was opened, three drops of Db3 (developer solution) were added, and the LFD dipstick was inserted. Results were read after 3–5 minutes. A positive result is indicated by the presence of both the control (C) and test (T) lines; a negative result shows only the C line; any other pattern, including the absence of both lines, is considered invalid and the test must be repeated. Positive (DNA) and negative (mock) controls were included in each experimental run to ensure assay validity.

To minimize the risk of amplicon carryover contamination, all assays were performed following a strict unidirectional workflow with segregation of materials and physical zones. Sample preparation and reaction setup were conducted in a clean or pre-amplification room (Area 1), whereas amplification and all post-amplification steps -including tube opening, addition of the developer solution, and LFD reading- were confined to a physically separate zone (Area 2). Crucially, this setup does not require complex high-containment infrastructure; standard, simple independent spaces are sufficient for field or low-complexity implementation. To prevent cross-contamination, a strict one-way operational flow was maintained, restricting personnel and material movement from Area 2 back into the pre-amplification room without proper apparel change and decontamination protocols. Dedicated post-amplification bench and thermoblock cleaning was easily managed through routine 70% ethanol wipe-downs, supplemented by periodic chemical amplicon degradation using a 0.1 M NaOH/0.5% SDS solution.

### 2.2 Parasite strains and DNA templates

Analytical assays were performed using purified DNA from *Trypanosoma cruzi* reference strains corresponding to each of the six discrete typing units (DTUs): Sylvio X10 (TcI), JG1 (TcII), M6241 (TcIII), M4167 (TcIV), PAH179 (TcV), and CL Brener (TcVI) (Zingales and Bartholomeu, 2022; Besuschio et al., 2017). For exclusivity assays, 10 pg of DNA from *Trypanosoma rangeli* (strains SC-58 and Choachi), *Trypanosoma brucei*, *Leishmania mexicana*, and human genomic DNA were included. This mass represented up to a 100-fold excess compared to the *T. cruzi* DNA target, establishing a stringent condition to rule out even low-efficiency cross-reactions.

Parasite DNA was extracted using either the TELT method (Medina-Acosta and Cross, 1993) or standard phenol-chloroform protocols. All DNA templates were quantified using a NanoDrop 1000 spectrophotometer (Thermo Scientific, Waltham, MA, USA) to standardize initial concentrations prior to amplification assays.

For analytical validations, purified DNA or whole *T. cruzi* CL Brener trypomastigotes were artificially spiked into negative human blood samples and subsequently spotted onto Whatman or S&S 903 filter paper (neonatal screening cards) to generate dried blood spots (DBS), preserving the complete clinical matrix and mimicking real neonatal screening conditions.

### 2.3 Analytical sensitivity: limit of detection

Analytical sensitivity was evaluated using serial dilutions of *T. cruzi* CL Brener genomic DNA (ranging from 10 pg to 0.1 fg per reaction) and known numbers of trypomastigotes (100, 10, and 1 parasite per reaction), artificially spiked into DBS samples on Whatman or S&S 903 filter paper (neonatal screening cards). All assays were performed in the presence of the complete clinical matrix to ensure selectivity conditions. The limit of detection (LoD) was defined as the lowest concentration yielding positive results in all replicates.

### 2.4 Repeatability

Repeatability (intra-assay precision) was evaluated by two independent operators, who processed DBS samples artificially spiked with known numbers of *T. cruzi* CL Brener parasites (100, 10, 1, and 0 parasites per reaction), in five replicates per condition, across different days. Results were visualized by LFD readout as described in Section 2.1. Data are summarized in **Supplementary Table S2**.

### 2.5 Analytical specificity (inclusivity and exclusivity)

Analytical specificity was assessed by evaluating both inclusivity and exclusivity in DBS samples artificially spiked onto Whatman or S&S 903 filter paper (neonatal screening cards). Inclusivity testing was performed using 50 fg of *T. cruzi* DNA from strains representing each of the six DTUs (TcI–TcVI). Exclusivity was assessed by the absence of cross-reactivity with 10 pg of DNA from heterologous organisms: *Trypanosoma rangeli* strain SC-58, *Trypanosoma rangeli* strain Choachi, *Trypanosoma brucei*, *Leishmania mexicana*, and human genomic DNA. Results were visualized by LFD readout as described in Section 2.1.

### 2.6 Clinical proof-of-concept panel

The clinical proof-of-concept of the LAMP-LFD system was evaluated using a reference panel of 24 anonymized DBS samples, collected within the first 48 hours of life from neonates born to *T. cruzi* seropositive mothers, obtained from the routine neonatal screening program at Hospital de Agudos Durand (Buenos Aires, Argentina). The diagnostic status of these specimens (20 negative and 4 positive) was blinded to the LAMP operators and had been established according to the national reference diagnostic algorithm in effect at the time of sample collection. This algorithm combined parasitological examination (microhematocrit/microStrout) within the first months of life and a confirmatory serological pair at 10 months of age in cases with a negative parasitological result. A subsequent update to the national guidelines (Ministerio de Salud de la República Argentina, 2025b) has since incorporated qPCR as an additional diagnostic tool; however, this had not yet been implemented when this panel was assembled and was therefore not evaluated in the present study.

For sample processing, a single 5.9 mm diameter disc (equivalent to approximately 15 µL of whole blood) was punched from each card using a micro-puncher and loaded directly into the reaction tube, following the extraction-free workflow described in Section 2.1. Clinical diagnostic performance metrics (sensitivity, specificity, and overall accuracy) were calculated, and exact 95% confidence intervals (95% CI) were determined using the Clopper-Pearson method to appropriately reflect the sample size.

## 3 Results

### 3.1 Primer set selection and analytical sensitivity

To optimize the system, reaction conditions -including reagent concentrations, incubation temperatures, and primer sets- were iteratively screened across different sample matrices, progressing from purified DNA to parasite suspensions, and finally to blood-spiked neonatal screening cards. Following this systematic optimization (data not shown), the reaction composition, the SAT-targeting primer set, and an amplification temperature of 64 °C for 60 minutes using a dry bath incubator were established as the definitive operating conditions. Among the primer sets initially evaluated (**Supplementary Table S1**), the SAT-targeting set demonstrated the highest analytical sensitivity and specificity, and was therefore selected for all subsequent assay development. The optimized assay is performed by direct loading of a DBS disc into the reaction tube without prior nucleic acid extraction, eliminating a major operational step required by other molecular diagnostic approaches.

When evaluated using purified genomic DNA spiked into whole blood and dried onto filter paper to mimic the clinical matrix, the LAMP-LFD assay detected *T. cruzi* DNA down to 1 fg per reaction (0.01 parasite equivalents), yielding consistently positive results by both agarose gel electrophoresis and LFD (**Figures 1a, b**). Based on rigorous reproducibility assays, this 1 fg threshold was established as the definitive analytical limit of detection (LoD); lower concentrations (0.1 to 0.01 fg) yielded sporadic reactivity. Furthermore, in DBS samples prepared from whole blood spiked with known numbers of parasites, a detection limit as low as 1 parasite per reaction was achieved (**Figures 1c, d**), while negative controls remained consistently non-reactive throughout all evaluations. It should be noted that while the analytical sensitivity for purified gDNA reaches 1 fg/reaction (approx. 0.01 parasite equivalents) due to the multicopy nature of the target, the LOD for intact organisms is inherently biologically bounded at 1 parasite/reaction, representing the minimum indivisible cellular unit that can be spiked into a given sample.

**Figure 1:**
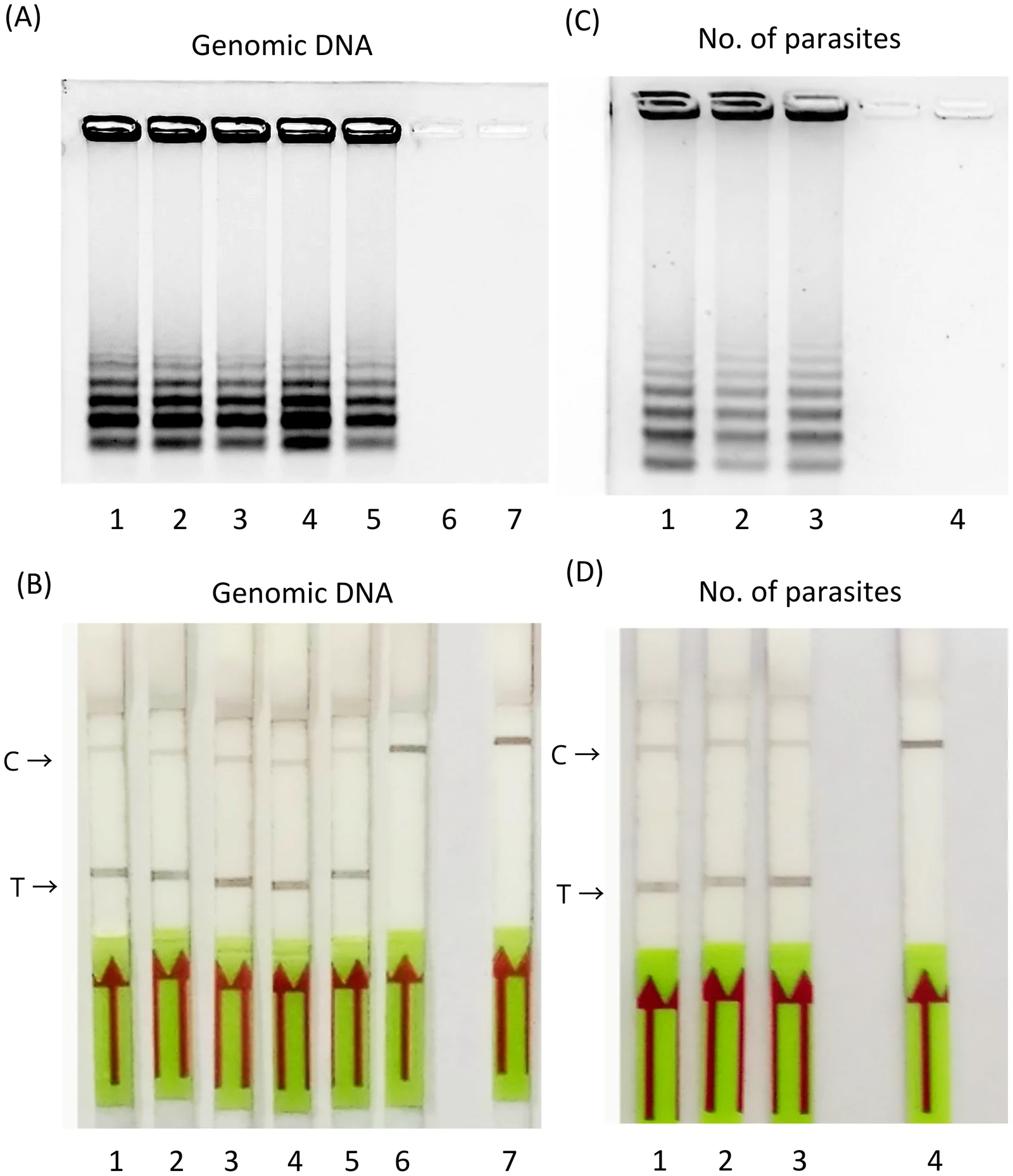
Analytical sensitivity of the developed LAMP-LFD assay under neonatal blood-matrix challenge. Serial dilutions of purified *T. cruzi* CL Brener (TcVI) genomic DNA **(a, b)** or known numbers of cultured parasites **(c, d)** were spiked into neonatal blood, spotted and dried onto newborn screening cards, and processed directly through the extraction-free workflow. The amplification reactions were incubated at 64 °C for 60 minutes. LAMP products were revealed by agarose gel electrophoresis **(a, c)** or by lateral flow dipstick (LFD) **(b, d)**. **(a, b)** Evaluation of genomic DNA sensitivity. Lane/Strip 1: 10 pg; lane/strip 2: 1 pg; lane/strip 3: 100 fg; lane/strip 4: 10 fg; lane/strip 5: 1 fg; lane/strip 6: 0.1 fg; lane/strip 7: NC (negative control). **(c, d)** Evaluation of parasite load sensitivity. Lane/Strip 1: 100 parasites per reaction; lane/strip 2: 10 parasites per reaction; lane/strip 3: 1 parasite per reaction; lane/strip 4: NC. LFD interpretation: C indicates the control line and T indicates the test line. Positive reactions display both C and T lines, whereas negative reactions show only the C line.

### 3.2 Analytical specificity and matrix compatibility

For inclusivity assays, samples representing all six *Trypanosoma cruzi* discrete typing units (DTUs TcI - TcVI) yielded consistently positive results, showing both Control (C) and Test (T) lines (**Figure 2**, lanes 1 - 6). Conversely, all exclusivity samples (**Figure 2**, lanes 7 - 11) -including the high-input DNA of two *T. rangeli* strains, *T. brucei*, *Leishmania mexicana*, and human genomic DNA- as well as the negative control (**Figure 2**, lane 12), yielded negative results with the C line only. These findings demonstrate robust analytical specificity across all *T. cruzi* lineages and a total absence of cross-reactivity with both phylogenetically related and unrelated organisms under stringent high-mass conditions.

**Figure 2:**
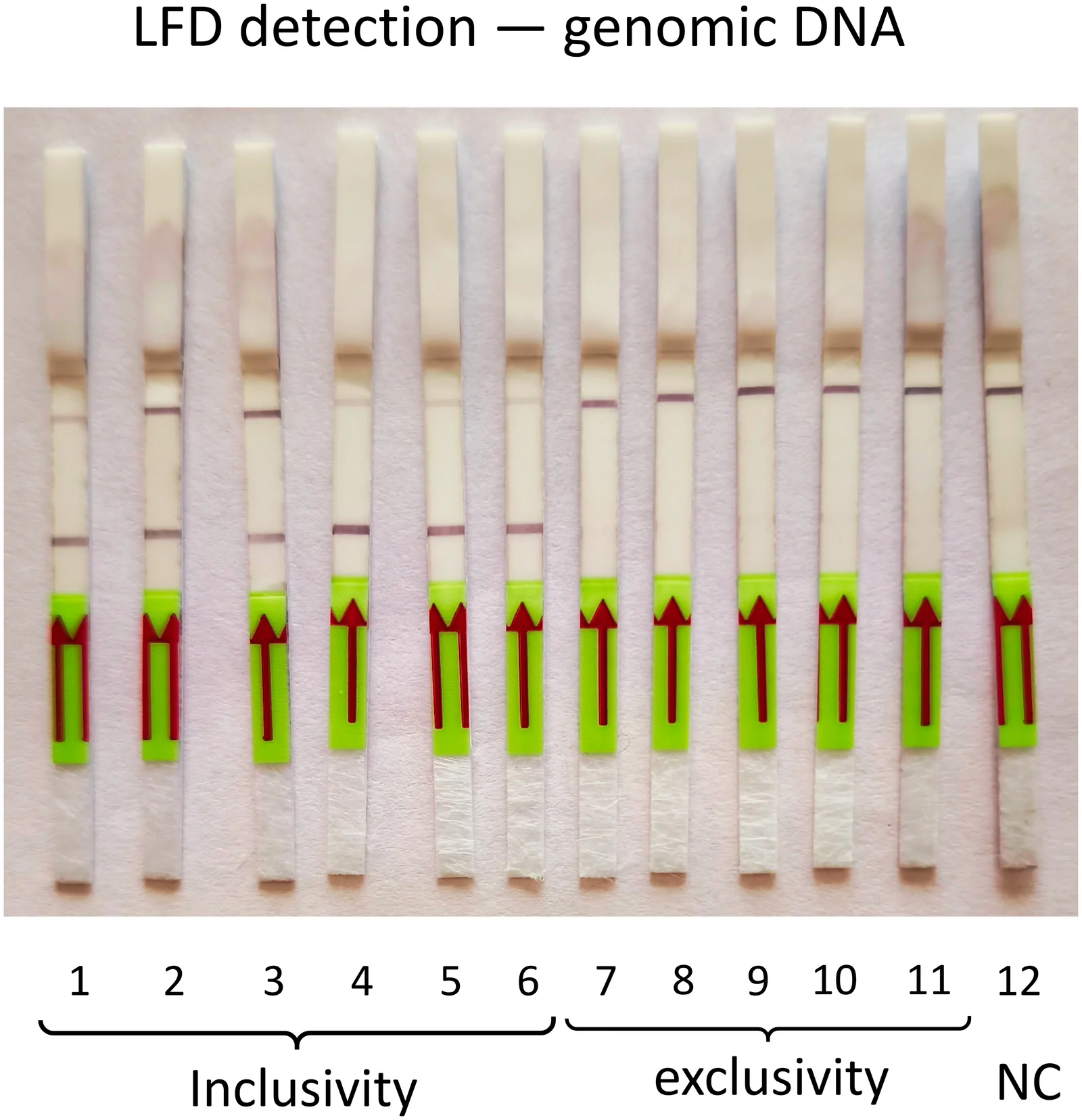
Analytical specificity and selectivity of the developed LAMP-LFD assay. LFD detection of LAMP products obtained from neonatal screening cards artificially spiked with genomic DNA from *T. cruzi* reference strains and non-target organisms, processed through the extraction-free workflow. Strips 1 - 6 (Inclusivity challenge) - *T. cruzi* genomic DNA (50 fg per reaction) representing all six discrete typing units (DTUs TcI - TcVI): strip 1, TcI (strain Sylvio X10); strip 2, TcII (strain JG1); strip 3, TcIII (strain M6241); strip 4, TcIV (strain M4167); strip 5, TcV (strain PAH179); strip 6, TcVI (strain CL Brener). Strips 7 - 11 (Exclusivity challenge) - Non-*T. cruzi* DNA templates (10 pg per reaction): strip 7, *T. rangeli* strain SC-58; strip 8, *T. rangeli* strain Choachi; strip 9, *Trypanosoma brucei*; strip 10, *Leishmania mexicana*; strip 11, human genomic DNA. Strip 12 (NC), Negative control. LFD interpretation: C indicates the control line and T indicates the test line. Positive reactions display both C and T lines, whereas negative reactions show only the C line.

Furthermore, the excellent matrix compatibility and analytical robustness of the developed kit were confirmed, as all sensitivity and specificity evaluations were conducted directly using whole blood dried onto neonatal screening filter paper, successfully reproducing the exact matrix conditions of clinical specimens without experiencing reaction inhibition or background interference.

### 3.3 Repeatability

Two independent operators testing DBS samples spiked with 100, 10, and 1 parasite per reaction achieved 5/5 positive results for all concentrations, and 0/5 for negative controls, yielding 100% concordance across all conditions and operators (**Supplementary Table S2**).

### 3.4 Clinical proof-of-concept

The developed LAMP-LFD assay correctly identified all 4 positive and all 20 negative specimens within the blinded DBS clinical proof-of-concept panel (n = 24), achieving 100% concordance with the definitive clinical status established by the national reference diagnostic algorithm (**Table 1**; **Supplementary Figure S1**). The assay successfully identified all positive samples, yielding a clinical sensitivity of 100% (4/4; 95% CI: 39.8% - 100%), and correctly ruled out all negative samples, resulting in a clinical specificity of 100% (20/20; 95% CI: 83.2% - 100%). Overall diagnostic accuracy was 100% (24/24; exact Clopper-Pearson 95% CI: 85.7% - 100%).

**Table 1:** Concordance between LAMP-LFD and reference diagnostic methods for congenital Chagas disease detection in neonatal DBS samples.

Diagnostic method	Positive	Negative	Total
Serology pair (10 months)	4	20	24
Microhematocrit/microStrout	1	23	24
LAMP-LFD (this study)	4	20	24

All 24 neonates were born to *T. cruzi* seropositive mothers (Hospital de Agudos Durand, Buenos Aires, Argentina). DBS samples were collected within the first 48 hours of life. The serology pair performed at 10 months of age served as the confirmatory reference test. No discordant results were observed between the LAMP-LFD assay and the serology pair. For detailed diagnostic performance metrics, please refer to the main text.

Notably, while 3 of the 4 positive cases had tested negative by conventional microhematocrit/microStrout examination during their first month of life (**Table 1**) -requiring a confirming serological pair at 10 months of age- the LAMP-LFD assay was capable of detecting the infection using only the screening cards collected -by law-, within the first 48 hours of life. These findings highlight the high diagnostic utility of molecular testing within an extremely simple isothermal format, positioning the LAMP-LFD assay as a highly promising screening tool for immediate, early detection of congenital *Trypanosoma cruzi* infection at birth.

## 4 Discussion

Congenital transmission remains a critical public health challenge, accounting for at least 15,000 new *Trypanosoma cruzi* infections annually in Latin America. In Argentina, where approximately 1,000 infected babies are born each year, this burden translates into a striking ratio of roughly 1 in 500 newborns nationwide, yet diagnostic coverage remains critically limited (PAHO, 2017; Ministerio de Salud de la República Argentina, 2022). This study demonstrates the robust analytical performance and clinical utility of our developed LAMP-LFD assay for the early detection of congenital Chagas disease. By enabling molecular-grade testing within an extremely simple isothermal format during the immediate neonatal period, this system addresses critical bottlenecks in current workflows, which frequently suffer from the low sensitivity of conventional microscopy, the high cost of qPCR, or the operational attrition of late serological testing, all of which drive high rates of patient loss to follow-up (Besuschio et al., 2017; Rivero et al., 2017; Rojas Panozo et al., 2024). Consequently, the LAMP-LFD assay emerges as a highly promising screening tool for early detection at birth and during the first months of life, particularly in resource-limited settings.

The inherent limitations of this study encompass two primary aspects: the reliance on artificially spiked blood samples for analytical validation and the small size of the blinded clinical panel (n = 24) utilized for the clinical proof-of-concept. Regarding the former, spiking protocols represent a widely accepted methodological standard to establish baseline analytical parameters under highly controlled conditions. For the latter, despite the preliminary scale of the clinical assay, the complete concordance achieved with the definitive clinical status underscores the high diagnostic utility of this isothermal test. Within this panel, the LAMP-LFD assay successfully detected all 4 confirmed positive neonates, whereas conventional microhematocrit/microStrout examination at birth identified only 1 of these 4 cases. This diagnostic gap is consistent with the well-documented limitations of parasitological methods, where parasite loads frequently fall below conventional microscopy thresholds (Carlier et al., 2019). Although the retrospective nature of this initial proof-of-concept panel precluded the direct correlation of assay readouts with standard qPCR Ct values, ongoing large-scale clinical validation studies are actively evaluating the assay’s diagnostic performance across stratified parasitemia levels.

A critical parameter in this direct-from-sample molecular test is balancing sample input with matrix inhibition. In this assay, a 5.9-mm-diameter DBS disc (∼15 μL of whole blood) was used. Although this blood volume accounts for 20% of the total reaction -a proportion that would severely inhibit standard PCR- the intrinsic tolerance of the Bst polymerase enables the system to buffer matrix components and function efficiently. This allowed the assay to incorporate a 5.9-mm disc, the largest size generated by the standard punching devices (3.2 mm, 4.7 mm, and 5.9 mm) commonly available at neonatal care facilities. Maximizing sample input is essential because the target DNA is confined within the parasite, resulting in a particulate rather than homogeneous distribution. Consequently, using the largest standard disc minimizes the stochastic risk of failing to capture the molecular template, thereby preserving the assay’s sensitivity. Considering that this 5.9-mm paper disc processes a blood sample volume of approximately 15 µL, our determined analytical LoD of 1 parasite per reaction corresponds to ~70 parasites/mL. This threshold is substantially below both the detection limit of the microhematocrit/microStrout technique (~1,000 parasites/mL) (Freilij et al., 1983) and the median parasitemia reported by direct micromethod at birth for infected neonates (1,700 parasites/mL) (Bua et al., 2013), validating the accuracy of our artificial spiking models. Furthermore, the system demonstrated complete analytical specificity -with no cross-reactivity against *Trypanosoma rangeli*, *Trypanosoma brucei*, *Leishmania mexicana*, or human DNA- and inclusivity across all six *T. cruzi* discrete typing units (DTUs TcI–TcVI) (Zingales et al., 2009; Bisio et al., 2021). Regarding the target’s biological variability, although the Sat-DNA copy number can vary naturally among *T. cruzi* DTUs (10^4^–10^5^ copies per haploid genome) (Elias et al., 2003), this does not measurably compromise the assay’s analytical sensitivity. Given that the analytical limit of detection reaches 1 fg of gDNA (approx. 0.01 parasite equivalents), even lineages with lower target abundance far exceed the required amplification threshold. This intrinsic sensitivity buffer is empirically supported by our inclusivity testing, where a fixed sub-genomic DNA input (50 fg) across all six DTUs yielded uniformly positive results (**Figure 2**). While specifically optimized here for congenital screening, this broad DTU coverage and high analytical tolerance to complex matrices suggest that the core technology could be readily adapted to other clinical and epidemiological scenarios characterized by high parasite burdens, such as acute or reactivated Chagas disease. The feasibility of such adaptations is strongly supported by the successful translation of this identical platform to vector surveillance through the direct testing of triatomine feces (Larocca et al., 2024), pending further validation in specific human clinical populations (Besuschio et al., 2020; Bisio et al., 2021).

From an operational standpoint, a major advantage of this assay is the direct processing of DBS specimens without prior nucleic acid extraction, driven by the intrinsic robustness of the *Bst* DNA polymerase synergized with the optimized Db1 master mix, facilitating direct *T. cruzi* amplification from the clinical matrix. While significant expertise has been developed across Latin America to implement LAMP for *T. cruzi* detection in screening cards, previous approaches have relied on DNA purification protocols (Rivero et al., 2017; Besuschio et al., 2017, Besuschio et al., 2020; Longhi et al., 2023; Gutierrez Guarnizo et al., 2025). By eliminating extraction kits, high-speed centrifuges, and specialized consumables, this streamlined, extraction-free workflow drastically reduces hands-on time and lowers costs. While long-term storage of the liquid reagents requires standard refrigeration (24 months at 4 °C), preliminary field-simulation studies indicate that full analytical performance is maintained for at least 15 days using basic portable cold chains (e.g., standard insulated coolers). This validates their operational suitability for decentralized workflows radiating from a central cold-storage hub. This operational simplicity aligns perfectly with Argentina’s National Newborn Screening Law (No. 26.279) and Chagas Disease Law (No. 26.281), as well as with public health policies in newborn screening programs and in vertical Chagas disease control established across other endemic and non-endemic countries. Because DBS cards are already systematically collected from newborns within the first 48 hours of life, our assay enables immediate molecular screening without adding clinical burdens or requiring new infrastructure, positioning it as a high-value complementary tool at birth. Such accessible molecular solutions remain essential, as therapeutic efficacy is highest during the neonatal period and is associated with cure rates that substantially exceed those achieved later in life (Sosa-Estani et al., 2012; Rojas Panozo et al., 2024).

When considering deployment in resource-limited settings, the choice of test readout strategy is a critical parameter. Real-time fluorescence-based LAMP offers a closed-tube format that inherently significantly reduces the risk of carryover contamination and enables kinetic monitoring, but it requires costly optical instrumentation (e.g., real-time thermal cyclers), posing a significant barrier in low-resource settings. Conversely, LFD detection provides high operational convenience and straightforward visual interpretation without specialized equipment, making it well suited for point-of-care applications in endemic regions; its main drawback is that reaction tubes must be opened post-amplification, entailing a risk of amplicon carryover. Recent studies have explored strategies to close this gap: real-time fluorescence approaches using specialized nucleases (e.g., RNase H II) and dual-labeled probes enable robust closed-tube detection (Zhang et al., 2023), while multi-enzymatic systems incorporating dUTP/UNG can be coupled with lateral flow platforms to actively prevent carryover contamination while preserving operational simplicity (Zhang et al., 2025). As noted above, our current LAMP-LFD assay manages carryover risk through spatial segregation, unidirectional workflows, and basic chemical decontamination rather than added enzymatic complexity. Nevertheless, implementing integrated closed-tube or contamination-control strategies remains an important goal for future improvements.

Significantly, the prototype evaluated in this study has culminated in an industrially transferred kit designed for extraction-free, qualitative *T. cruzi* detection. This kit, adopted by Laboratorio Pablo Cassará SRL (Argentina) under the name CHAGAS NEOKIT TecnoAMI, has been granted regulatory approval by ANMAT (Registration Code PM-40-13). To our knowledge, this represents the first LAMP-based diagnostic kit approved by a national regulatory authority for Chagas disease screening, marking a milestone in the translation of academic R&D into accessible public health tools. Ultimately, the direct compatibility of this system with existing infrastructure establishes this simplified molecular assay as a highly viable and scalable option for integration within national neonatal screening programs, both in Argentina and in other endemic or non-endemic regions facing vertical *T. cruzi* transmission.

## Data Availability

The original contributions presented in the study are included in the article/**Supplementary Material**, further inquiries can be directed to the corresponding author.
