# Supplementary material for: Extraction-free LAMP-LFD assay for early diagnosis of congenital Chagas disease directly from neonatal dried blood spots
Source: Front Cell Infect Microbiol. 2026 Oct 1;16:1934037. doi: 10.3389/fcimb.2026.1934037 (PMC13668752; doi:10.3389/fcimb.2026.1934037)
Supplement: Supplementary file 1 [file DataSheet1.pdf]

Supplementary Material

1 Supplementary Figures and Tables

1.1 Supplementary Tables

Supplementary Table S1. LAMP primer sets evaluated for the detection of *Trypanosoma cruzi*. All evaluated primer sets were synthesized with 5'-biotin-labeled FIP and 5'-FAM-labeled BIP primers to enable lateral flow dipstick (LFD) visual readout. The SAT primer set was selected for final assay integration based on its optimal analytical sensitivity under high-matrix challenge.

Target region	Type	Name	Sequence (5'→3')	nt	Label	Molecular target	GenBank	Analytical sensitivity
**SAT**	FIP	SAT-FIP	CACTCGGCTGATCGTTTTCGACCCCCATCATTCATAATTGGA	42	Biotin	Nuclear satellite DNA 195 bp	AY519985-AY520098	**High**
**SAT**	BIP	SAT-BIP	GGCAAGAGCTCGCGAAATTCCACACACTGGACACCAAA	38	FAM	Nuclear satellite DNA 195 bp	AY519985-AY520098	
**SAT**	LF	SAT-LF	TGCATCACATGTTGTGGTCTA	21	-	Nuclear satellite DNA 195 bp	AY519985-AY520098	
**SAT**	LB	SAT-LB	TCCAAGCAGCGGATAATTCA	20	-	Nuclear satellite DNA 195 bp	AY519985-AY520098	
**SAT**	F3	SAT-F3	ACAGAGAGTGCCTCTGA	17	-	Nuclear satellite DNA 195 bp	AY519985-AY520098	
**SAT**	B3	SAT-B3	GTCAATATCTGTTTGCG	17	-	Nuclear satellite DNA 195 bp	AY519985-AY520098	
**24S-2**	FIP	24S2-FIP	CGGTGTAACGTTGTGCTCAGTAGGAAGGATTTCGTGCCAA	40	Biotin	24S-alpha rRNA (D7 domain)	M28885.1	**Moderate**
**24S-2**	BIP	24S2-BIP	TGTTAGGAAAGTGAAGGTGCGTTGAAGAGAAGGAAAGAGAGGTA	44	FAM	24S-alpha rRNA (D7 domain)	M28885.1	

**24S-2**	LF	24S2-LF	TCCGAACAACTC GTGTACG	19	-	24S-alpha rRNA (D7 domain)	M28885.1	
**24S-2**	LB	24S2-LB	GACAGTGTGGGA GTCCTTT	19	-	24S-alpha rRNA (D7 domain)	M28885.1	
**24S-2**	F3	24S2-F3	CAGATCTTGGTTG GCGTAG	19	-	24S-alpha rRNA (D7 domain)	M28885.1	
**24S-2**	B3	24S2-B3	CAGAATGGCCGA ACAGTAA	19	-	24S-alpha rRNA (D7 domain)	M28885.1	
**kDNA -1**	FIP	KDNA1-FIP	GGTTCGATTGGGG TTGGTGTGCATCA TAGCATACCAATC TGT	42	Biotin	kDNA minicircle - conserved region	Sturm et al. 1989	**Low**
**kDNA -1**	BIP	KDNA1-BIP	CGTAAACGCACC CCATTTTCGGAGG GGCGTTCAACTTT T	39	FAM	kDNA minicircle - conserved region	Sturm et al. 1989	
**kDNA -1**	LF	KDNA1-LF	AGGAGTTGTGTTT GGTGGG	19	-	kDNA minicircle - conserved region	Sturm et al. 1989	
**kDNA -1**	LB	KDNA1-LB	CCAAATAATGTAC GGGGGAGAT	22	-	kDNA minicircle - conserved region	Sturm et al. 1989	
**kDNA -1**	F3	KDNA1-F3	CTGTTTATACACT GTCACATCC	22	-	kDNA minicircle - conserved region	Sturm et al. 1989	
**kDNA -1**	B3	KDNA1-B3	TGCACTTTGTGGG TTTCT	18	-	kDNA minicircle - conserved region	Sturm et al. 1989	
**kDNA -2**	FIP	KDNA2-FIP	CGAAAATGGGGT GCGTTTACGACTC CTTATATTACACC AACCC	43	Biotin	kDNA minicircle - conserved region	Sturm et al. 1989	**Low**
**kDNA -2**	BIP	KDNA2-BIP	AAAGTTGAACGC CCCTCCCTGCACT TTGTGGGTTTCT	37	FAM	kDNA minicircle - conserved region	Sturm et al. 1989	
**kDNA -2**	LF	KDNA2-LF	GGAGGTGGGGTT CGATTG	18	-	kDNA minicircle -	Sturm et al. 1989	

						conserved region		
**kDNA-2**	LB	KDNA2-LB	TCTAGTTTTTCTGGGCCACAA	21	-	kDNA minicircle - conserved region	Sturm et al. 1989	
**kDNA-2**	F3	KDNA2-F3	CTGTAACCCACCAAACACA	19	-	kDNA minicircle - conserved region	Sturm et al. 1989	
**kDNA-2**	B3	KDNA2-B3	GTATGTATTTATCTATGTTGTG	22	-	kDNA minicircle - conserved region	Sturm et al. 1989	
**kLbQm**	FIP	kLbQm-FIP	AGTTGTGTTTGGTGGGTTACAGACAATATACTGTAACACCATTAATGC	48	Biotin	Conserved hypothetical protein	AJ748068.1	**Low**
**kLbQm**	BIP	kLbQm-BIP	CCGTAAACGCACCCCATTTTACTTTTTGGGGAAAATTCATG	42	FAM	Conserved hypothetical protein	AJ748068.1	
**kLbQm**	LF	kLbQm-LF	CAATATACTGTAACACCATTAATGC	25	-	Conserved hypothetical protein	AJ748068.1	
**kLbQm**	LB	kLbQm-LB	ACTTTTTGGGGAAAAATTCATG	22	-	Conserved hypothetical protein	AJ748068.1	
**kLbQm**	F3	kLbQm-F3	TCCATACTATACACATAAACCATAG	25	-	Conserved hypothetical protein	AJ748068.1	
**kLbQm**	B3	kLbQm-B3	AGAAAGTTTTGTTTTGGGAGG	21	-	Conserved hypothetical protein	AJ748068.1	
**AK-A**	FIP	AK-A-FIP	TGAGCGTGGCGCCCATTTCCATCGAAGGAGGTGTTTGA	38	Biotin	Arginine kinase	AF070451.1	**No amplification**
**AK-A**	BIP	AK-A-BIP	TCATCCAGTCGGGCGTGGAGAAGGTGTACGACTCGGCG	38	FAM	Arginine kinase	AF070451.1	
**AK-A**	LF	AK-A-LF	CTCACGAAGGAGGTGTTTGA	20	-	Arginine kinase	AF070451.1	
**AK-A**	LB	AK-A-LB	AAGGTGTACGACTCGGCG	18	-	Arginine kinase	AF070451.1	

**AK-A**	F3	AK-A-F3	GCCACTCGCTCCT CAAGA	18	-	Arginine kinase	AF070451. 1	
**AK-A**	B3	AK-A-B3	TCGATGATCGGGT CGAAGAG	20	-	Arginine kinase	AF070451. 1	
FIP: Forward Inner Primer (labeled with biotin). BIP: Backward Inner Primer (labeled with FAM). LF/LB: Loop primers. F3/B3: Outer primers. nt: nucleotide length. Analytical sensitivity: qualitative classification based on the lowest DNA concentration consistently detected in DBS samples: High = detection at ≤1 fg/reaction; Moderate = detection at 10-100 fg/reaction; Low = detection only at ≥1 pg/ reaction; No amplification = no product detected; Not evaluated = not tested.								

Supplementary Table S2. Repeatability of the developed LAMP-LFD assay evaluated by two independent operators. Assay precision was assessed using neonatal screening cards artificially spiked with known numbers of *T. cruzi* CL Brener (TcVI) parasites and processed directly through the extraction-free workflow.

**Number of parasites (per reaction)**	**Operator 1**		**Operator 2**		**Concordance**
	**Positive / Total**	**Result**	**Positive / Total**	**Result**	
**100 parasites**	**5/5**	**Positive**	**5/5**	**Positive**	**100%**
**10 parasites**	**5/5**	**Positive**	**5/5**	**Positive**	**100%**
**1 parasite**	**5/5**	**Positive**	**5/5**	**Positive**	**100%**
**0 parasites (NC)**	**0/5**	**Negative**	**0/5**	**Negative**	**100%**
NC: negative control. n = 5 per operator. DBS samples spiked with *T. cruzi* CL Brener (TcVI) on Whatman 903 neonatal screening cards.					
