# Supplementary material for: Extraction-free LAMP-LFD assay for early diagnosis of congenital Chagas disease directly from neonatal dried blood spots
Source: Front Cell Infect Microbiol. 2026 Oct 1;16:1934037. doi: 10.3389/fcimb.2026.1934037 (PMC13668752; doi:10.3389/fcimb.2026.1934037)
Supplement: Supplementary file 2 [file DataSheet2.pdf]

Supplementary Material

1 Supplementary Figures and Tables

1.1 Supplementary Figures

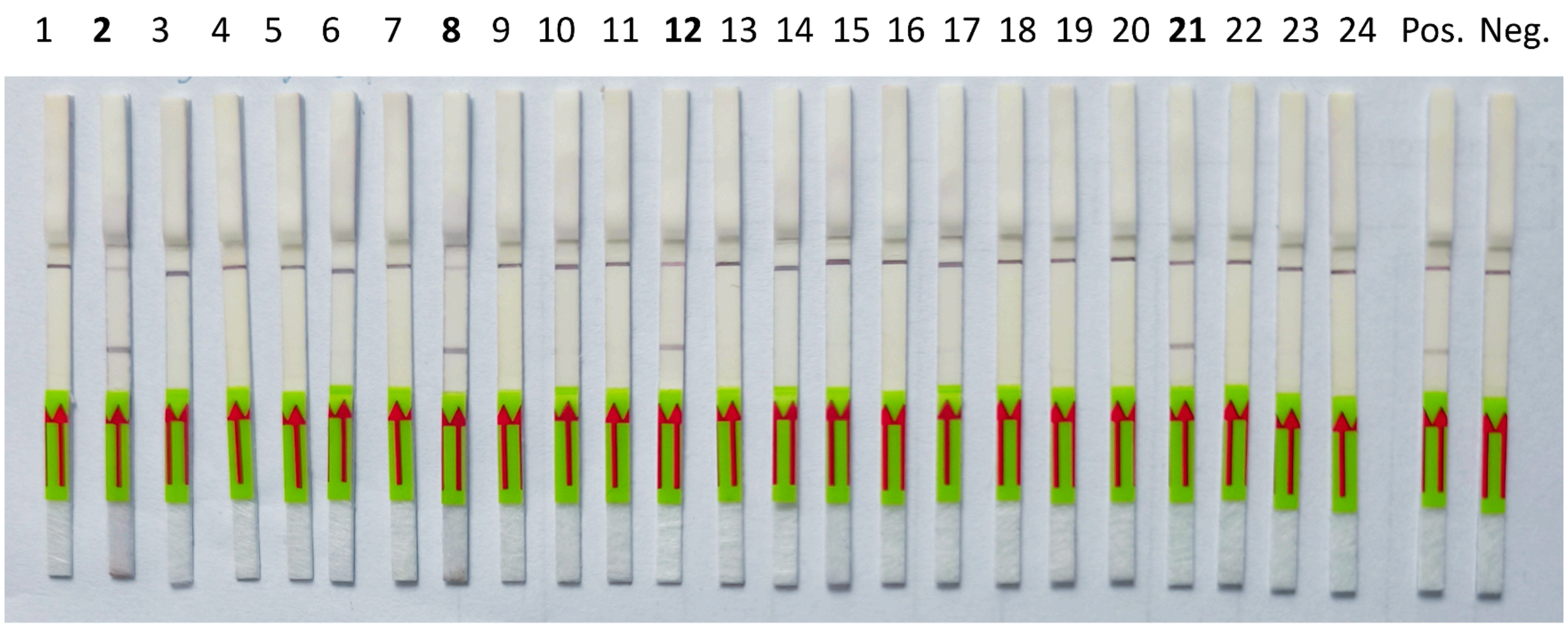

Supplementary Figure S1. Clinical Proof-of-Concept of the developed LAMP-LFD assay using a blinded neonatal panel. LFD detection of LAMP products obtained directly through the extraction-free workflow from clinical DBS samples collected within the first 48 hours of life from 24 neonates born to *T. cruzi*-seropositive mothers (routine Neonatal Screening Program, Hospital de Agudos Durand, Buenos Aires, Argentina). Specimens were anonymized and assigned sequential numbers to protect patient identity. Strips 2, 8, 12, and 21 (bold numbers) indicate positive screening results. Pos (Positive Control): Reaction containing 100 fg of purified *T. cruzi* genomic DNA; Neg (Negative Control): Reaction containing amplification reagents and water instead of sample matrix. LFD interpretation: C indicates the control line and T indicates the test line. Positive reactions display both C and T lines, whereas negative reactions show only the C line.
